# Associations between over-the-counter analgesics usage and symptoms of anxiety and depression in adolescents: a network analysis

**DOI:** 10.1186/s12888-024-05802-3

**Published:** 2024-05-15

**Authors:** Elise Solbu Roalsø, Sandra Klonteig, Brage Kraft, Siv Skarstein, Marianne Aalberg, Rune Jonassen

**Affiliations:** 1 Faculty of Health Sciences, Oslo Metropolitan University, Oslo, Norway; 2 Advanced Health Intelligence and Brain-Inspired Technologies (ADEPT), Oslo Metropolitan University, Oslo, Norway; 3 Division of Mental Health Services, Akershus University Hospital, Akershus, Norway; 4 Division of Psychiatry, Diakonhjemmet Hospital, Oslo, Norway

**Keywords:** (Over-the-counter) analgesics, Acetaminophen, Ibuprofen, Anxiety, Depression, Pain, Adolescents, Gender/sex differences, Network analysis

## Abstract

**Background:**

The use of over-the-counter analgesics (OTCA) is common among adolescents and has been linked with increased symptoms of anxiety and depression. However, little is known about which specific symptoms are most strongly connected to OTCA usage. The current study assessed which anxiety and depression symptoms were most closely associated with OTCA usage in a large sample of adolescents and examined whether this differed across genders.

**Method:**

The present study was based on data from 626,581 participants from the Ungdata survey in Norway. Associations between OTCA and anxiety and depression symptoms were examined using network analysis. Non-regularized partial-correlation networks were constructed to estimate the conditional dependent relations between the use of OTCA and symptoms while controlling for pain. Gender-specific networks were created for comparison.

**Results:**

OTCA usage was associated with most symptoms, even after controlling for pain, with the strongest associations with “sleep problems”, “stiff or tense”, “everything is a struggle” and “suddenly scared”. There were some gender differences, showing that “sleep problems” and “hopeless” were more strongly related to OTCA usage in females, whereas “stiff or tense” was more strongly related to OTCA usage in males.

**Conclusion:**

Overall, the somatic symptoms of anxiety and depression displayed the strongest associations with OTCA usage. When examining the gender-specific networks, both showed similar trends, although males exhibited slightly stronger associations between OTCA usage and somatic symptoms.

**Supplementary Information:**

The online version contains supplementary material available at 10.1186/s12888-024-05802-3.

## Background

The use of over-the-counter analgesics (OTCA), such as paracetamol (acetaminophen) and ibuprofen, is highly recurrent and increasing among adolescents, particularly among young girls [1, 2]. On-label usage of OTCA implies using analgesics to alleviate pain symptoms. Adolescents with high levels of psychiatric symptoms report more frequent occurrences of pain, such as headaches, abdominal pain and chronic pain [3, 4]. Remarkably, even after adjusting for pain symptoms, adolescents with elevated psychiatric symptoms still show more frequent analgesic usage [5, 6]. One question that emerges is whether adolescents use OTCA off-label to alleviate psychological and emotional distress.

There are a few experiments indicating that OTCA usage may lead to a decrease in depression [7] and anxiety [8]. However, these studies, along with others we have reviewed, approaches anxiety and depression as distinct constructs rather than analyzing individual symptoms [2, 5, 6]. Given the potential complex relationship between OTCA usage and anxiety and depression, we deem it crucial to explore the relationship between OTCA usage and several specific symptoms of anxiety and depression.

Pain is often accompanied by somatic symptoms such as poor sleep, tension or stiffness, fatigue, and changes in appetite [9]. These symptoms are also commonly associated with psychological complaints. Anxiety and depression are among the most prevalent classes of psychiatric disorders in adolescents [10]. They involve a wide array of psychological, physiological and cognitive symptoms, and have long been acknowledged as multisystem disorders affecting, and affected by, both mental and physical health [11, 12]. Both conditions result in substantial disability and pose massive and increasing global health challenges [13].

Several studies have demonstrated a strong correlation between measures of anxiety and depression across both general and clinical populations [14, 15]. The two constructs likely encompass a diverse range of diagnoses with partially overlapping causes and underlying mechanisms, leading to high rates of comorbidity [16]. The substantial overlap between anxiety and depression has prompted numerous theories and models attempting to explain their intricate relationship, with most of these suggesting both shared and distinct characteristics and symptoms between them (e.g., [17–19]). Still, research on anxiety and depression tend to treat them as distinct conditions [20].

Studies indicate that the presence and co-occurrence of pain and anxiety and depression are more prevalent in females than males [6, 21, 22], and that young women in particular might use OTCA as a tool to downregulate feelings of distress [2, 23]. This is further supported by research indicating gender differences in physical pain perception and response to analgesic usage [24, 25] and experiences of social pain [26]. Considering this, it may be that OTCA usage is more strongly linked with anxiety and depression symptoms in females.

### A network approach to OTCA usage and anxiety and depression symptoms

The network theory of mental disorders advocates for psychological constructs to be understood as overlapping clusters of heterogeneous symptoms (e.g., [20, 27–31]). These clusters are theorized to emerge from the causal interactions between symptoms, rather than being caused by an underlying construct [32]. Using network analysis, researchers can address this complexity by examining the correlation among specific symptoms [28], as well as other relevant psychological and medical constructs [31].

Based on this perspective, we conceptualize the association between OTCA usage and symptoms of anxiety and depression as a potential causal system (a network), cutting across traditional diagnostic boundaries [33]. Given that the on-label indication for OTCA usage is pain, OTCA usage would likely be directly associated with headaches in such a network. However, headaches may also be associated with OTCA usage indirectly because headaches may lead to excessive worrying and sad mood, which may lead to tension and stiffness, which in turn leads to OTCA usage. In this case, the association between OTCA usage and anxiety and depression is linked through *somatic symptoms* shared between pain and psychological distress. On another note, because previous studies have shown that OTCA is used off-label to relieve distress and social pain [34–36], OTCA usage may also be directly linked with affective and cognitive symptoms.

The aforementioned examples illustrate the potential intricate relationship between OTCA usage and anxiety and depression symptoms. Using the network approach, the present study examines this relationship in a population-wide sample of adolescents in Norway. In this specific population, is OTCA usage correlated to the same extent with all symptoms, or with specific symptoms? In the case of the latter, which symptoms are most closely related with OTCA usage?

### The present study

The primary aim of the present study was to examine whether OTCA usage in adolescents is associated with specific symptoms of anxiety and depression, using network analysis. Given the absence of prior research assessing distinct symptom associations with OTCA usage, we employed an exploratory approach. As a secondary aim we examined whether the associations between OTCA usage and specific symptoms differed across genders. We hypothesised that symptoms of anxiety and depression would be more prominently associated with OTCA usage in females compared to males (e.g., 2, 6, 46–51).

## Method

### Sample collection

We obtained data from an annual nationwide survey of adolescents in Norway (“Ungdata”). The Ungdata surveys are administrated online during school hours across all municipalities, ensuring representation from both urban and rural areas in Norway. The Norwegian Social Welfare Research Institute (NOVA) manages a national database containing all conducted surveys. For this study, we included all junior and senior high school students aged 12–19 years from municipalities that conducted the Ungdata survey between 2014 and 2019 (*N* = 626,581). Surveys conducted before 2014 did not include items related to OTCA. Pain-related items underwent a substantial revision in 2019, leading us to exclude all data collected after 2019. By doing this, we also excluded data from the time of the COVID-19 pandemic, which could potentially have influenced the observed relationships. In the gender-specific analysis, we excluded all participants with missing gender data, which amounted to 19,010 participants (3%).

### Assessments

#### Over-the-counter analgesics

Participants were asked how often they had used OTCA (paracetamol, ibuprofen, etc.) over the past month. The response options were: 1) Never, 2) Less than once a week, 3) At least weekly, 4) Several times a week and 5) Daily.

#### Anxiety and depression symptoms

Ungdata’s main module, titled “Mental Health Issues”, which is administered annually, is based on a combination of the Hopkins Symptom Checklist (HSCL-10) ( [37] and the Depression Mood Inventory [38], encompassing a total of nine items. Additionally, Ungdata has included a question regarding loneliness, resulting in a total of 10 items used to measure anxiety and depression symptoms in our analysis. These items included: 1) Felt that everything is a struggle, 2) Had sleep problems, 3) Felt unhappy, sad or depressed, 4) Felt hopeless about the future, 5) Felt stiff or tense, 6) Worried too much about things, 7) Felt lonely, 8) Suddenly felt scared for no reason, 9) Felt constant fear or anxiety and 10) Been nervous or felt uneasy. Participants were asked to rate these symptoms during the preceding week on a 4-point scale with the following response options: 1) Not been affected at all, 2) Not been affected much, 3) Been affected quite a lot and 4) Been affected a great deal.

#### Pain symptoms

The assessment of pain, which consisted of four items, asked about 1) Headache, 2) Neck and shoulder pain, 3) Joint and muscle pain, and 4) Abdominal pain in the past month. The response options were 1) Never, 2) A few times, 3) Many times and 4) Daily.

#### Statistical analyses

We used network analysis [28] to examine the associations between symptoms and OTCA usage. Results are presented as graphical networks consisting of nodes representing the study variable and edges between nodes representing conditional independence relations (partial correlations) between them. In line with guidelines [39], node variables were nonparanormally transformed using the R package *huge* [40]*.* Potential node redundancy among the symptoms due to topological overlap was ruled out using the Goldbricker function in networktools [41]. Networks were then estimated by computing unregularized Gaussian Graphical Models (GGMs) using the ggmModSelect function in qgraph (version 1.5 [42];). This procedure has been demonstrated to exhibit strong specificity in large samples, with better psychometric properties than for example graphical LASSO [43, 44]. We used full information maximum likelihood estimation, which takes into account all observed data, including missing data (17.4% of data points were missing). Specifically, ggmModSelect employs an iterative process to select the optimal GGM based on an extended Bayesian information criterion [44]. This procedure eliminates spurious associations (edges) attributable to the influence of other nodes in the network and shrinks trivially small associations to zero, thereby reducing the number of potentially *false positive* edges from the network and producing a sparse graph comprising only the strongest edges. We defined three communities in line with our three node categories (i.e. OTCA, symptoms, pain) to identify nodes bridging between them. Nodes which served as bridges in connecting the predefined communities (i.e., bridge nodes) were identified by computing the Bridge Expected Influence (BEI) index using the R package *networktools* [45, 46]. Accuracy of the network estimates was assessed using the *bootnet* R package version 1.5.1 [47] with 1000 bootstrap samples.

The analysis consisted of three stages. In the first stage, we estimated a network consisting of anxiety and depression symptoms and OTCA usage. This network highlights associations between specific symptoms and analgesic usage. In the second stage, we added the pain nodes, thereby controlling for the effect of pain. Notable edges and bridge nodes were identified at this stage. In the final stage, we computed one network for females and one network for males and examined the differences between them by head-to-head comparisons of symptom-OTCA edges (i.e., edge weights) and BEI of the OTCA node, using Holm–Bonferroni correction for multiple comparisons. Differences in edge weights and BEI between the networks were evaluated statistically using the R package *network comparison test* (NCT) version 2.2.1 [48] with 1000 iterations.

## Ethical considerations

The study was conducted in accordance with the Declaration of Helsinki. The adolescents were asked to provide informed consent prior to participating. Parents were informed about the study in advance and were given the opportunity to reserve their child from participation. The study was approved by The Norwegian National Committee for Research Ethics in the Social Sciences and the Humanities (NESH) (reference number: 2021/121) and the Norwegian Centre for Research Data (reference number: 821474). Data were gathered anonymously and subsequently analyzed by impartial researchers who were not involved in the data collection.

## Results

### Sample characteristics

The total sample consisted of 303,059 (48.4%) females and 304,512 (48.6%) males. 19,010 (3%) did not report gender and were thus excluded from the gender-specific network analyses. The proportion of weekly OTCA users was 23.4% among females and 11.2% among males. The majority of the sample (62.5%) attended junior high school (ages 12–16), while 37.5% were enrolled in senior high school (ages 16–19).

### Associations between OTCA usage and anxiety and depression symptoms

**Fig. 1:**
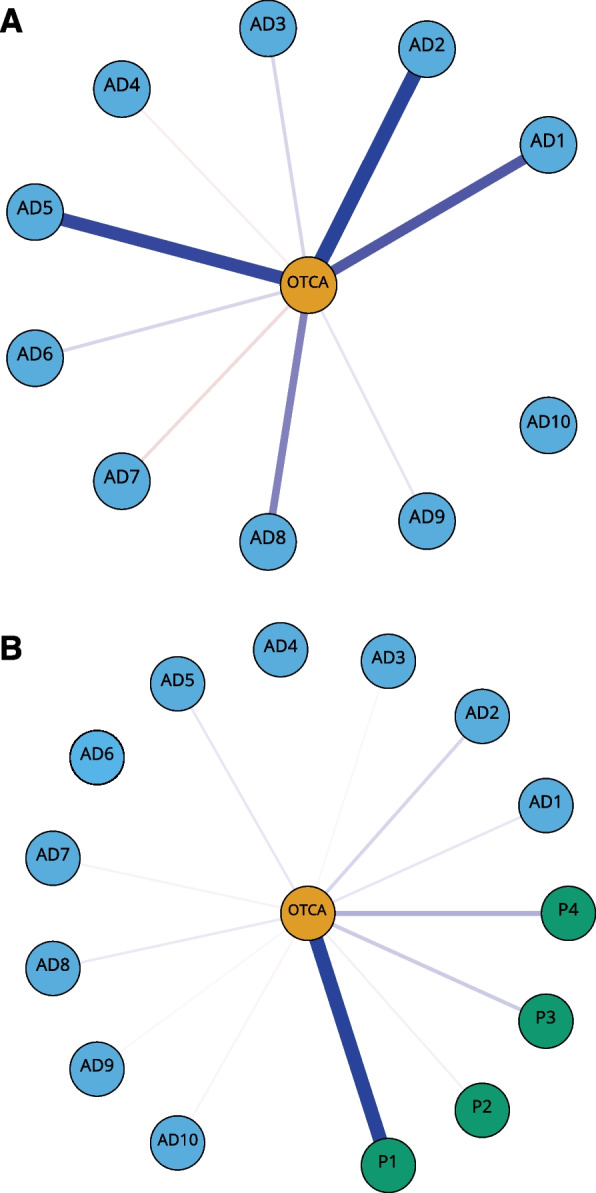
**A** Non-regularized partial-correlation network of anxiety and depression symptoms and OTCA usage. Blue edges represent positive and red edges represent negative partial correlations. The OTCA node is placed in the centre of a circle consisting of the remaining variables. To facilitate interpretation, all other edges than those connected to OTCA have been omitted from the graphical presentation. The thickness and brightness of an edge indicate association strength. Legends: OTCA: Paracetamol, ibuprofen or similar, AD1: Felt that everything is a struggle (edge weight: 0.07), AD2: Had sleep problems (0.1), AD3: Felt unhappy, sad or depressed (0.02), AD4: Felt hopeless about the future (− 0.01), AD5: Felt stiff or tense (0.09), AD6: Worried too much about things (0.02), AD7: Felt lonely (− 0.01), AD8: Suddenlty felt scared for no reason (0.05), AD9: Felt constant fear or anxiety (0.01), AD10: Been nervous or felt uneasy (0.0). **B** Non-regularized partial-correlation network with pain symptoms as covariates. Blue edges represent positive and red edges represent negative partial correlations. The OTCA node is placed in the centre of a circle consisting of the remaining variables. To facilitate interpretation, all other edges than those connected to OTCA have been omitted from the graphical presentation. The thickness and brightness of an edge indicate association strength. Legends: OTCA: Paracetamol, ibuprofen or similar, AD1: Felt that everything is a struggle (edge weight: 0.03), AD2: Had sleep problems (0.05), AD3: Felt unhappy, sad or depressed (0.01), AD4: Felt hopeless about the future (0.0), AD5: Felt stiff or tense (0.03), AD6: Worried too much about things (0.0), AD7: Felt lonely (− 0.01), AD8: Suddenly felt scared for no reason (0.02), AD9: Felt constant fear or anxiety (0.01), AD10: Been nervous or felt uneasy (− 0.01), P1: Headache (0.3), P2: Neck and shoulder pain (0.01), P3: Joint and muscle pain (0.06)*,* P4: Abdominal pain (0.09)

Associations between OTCA usage and symptoms are presented in Fig. 1A. Here, nodes represent the study variables, and the edges represent associations between them. Thicker edges represent stronger associations. In line with our objective on symptom-OTCA associations, only edges that were connected to OTCA were visualized. Note that the rest of the edges were accounted for in all the networks’ analyses, and only omitted in the graphical presentation in the figures. Networks visualizing all edges can be found in the Supplementary Materials.

OTCA usage was most strongly associated with “sleep problems” (edge strength = 0.10), “stiff or tense” (0.09), “everything is a struggle” (0.07) and “suddenly scared” (0.05). When adding pain variables to the model (Fig. 1B), results showed that OTCA usage was most strongly associated with “headache” (0.29) and “abdominal pain” (0.08). There were still associations between OTCA usage and most anxiety and depression symptoms, but the associations were weaker.

**Fig. 2:**
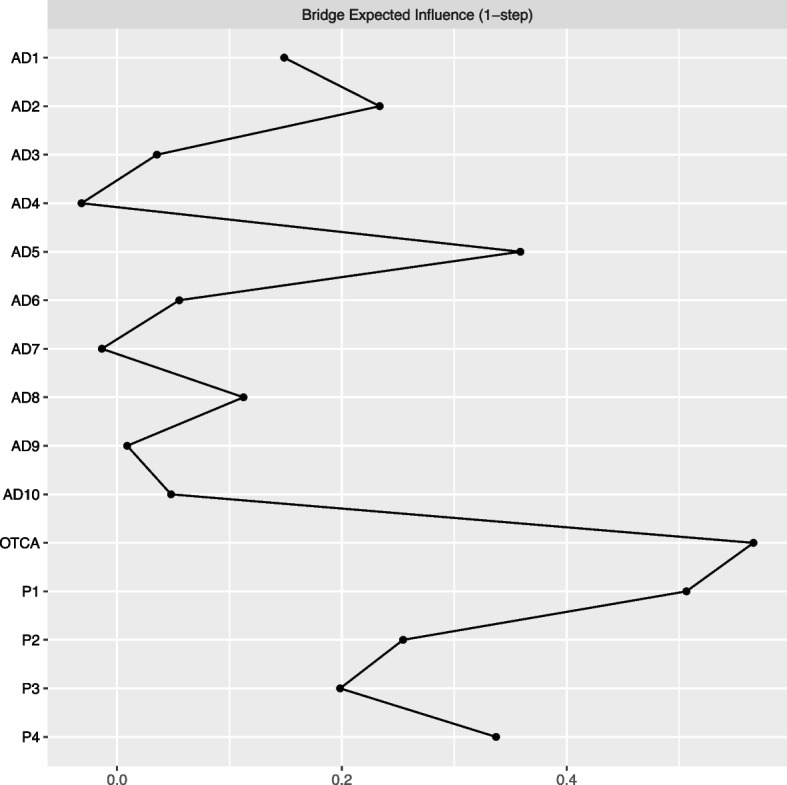
Bridge expected influence for Network 2. We defined three communities (i.e. OTCA + symptoms + pain) to check for nodes bridging between them. Bridge expected influence 1-step refers to the sum of the value (positive or negative) of all edges connecting node A to all nodes not in the same community as node A [46]. Legends: OTCA: Paracetamol, ibuprofen or similar, AD1: Felt that everything is a struggle, AD2: Had sleep problems, AD3: Felt unhappy, sad or depressed, AD4: Felt hopeless about the future, AD5: Felt stiff or tense, AD6: Worried too much about things, AD7: Felt lonely, AD8: Suddenly felt scared for no reason, AD9: Felt constant fear or anxiety, AD10: Been nervous or felt uneasy. Blue edges represent positive and red edges represent negative partial correlations. The OTCA node is placed in the centre of a circle consisting of the remaining variables. The thickness and brightness of an edge indicate association strength, P1: Headache, P2: Neck and shoulder pain, P3: Joint and muscle pain*,* P4: Abdominal pain

Bridge centrality indices are presented in Fig. 2 and signify which nodes are most central in explaining associations between OTCA usage, symptoms, and pain. Among the symptoms, “stiff or tense” was the most important bridge node, while “headache” was the most important bridge node among the pain variables.

### Gender differences

**Fig. 3:**
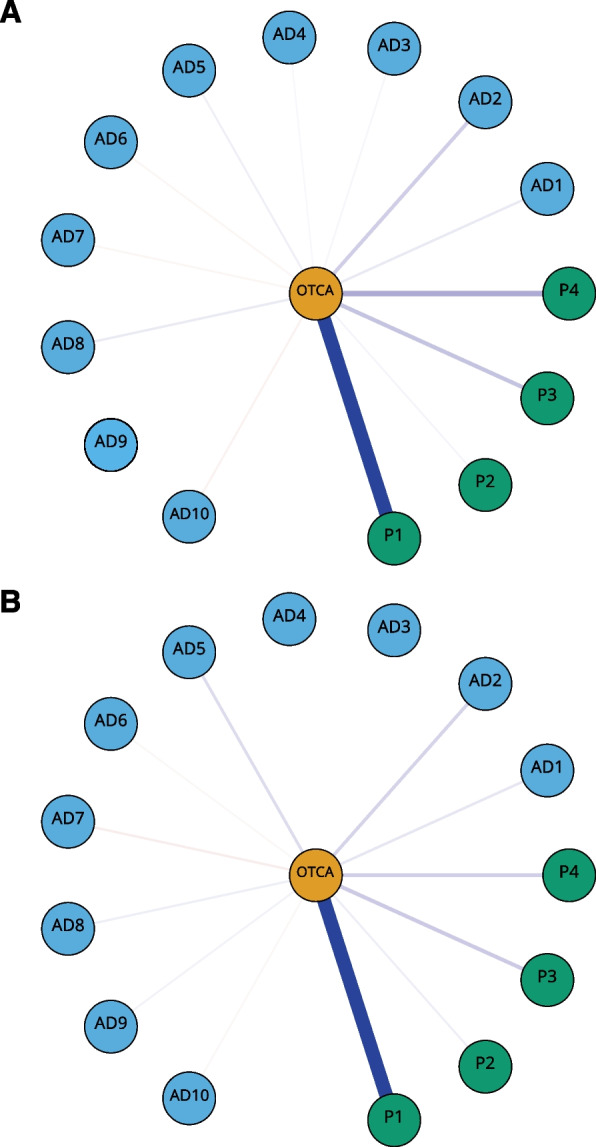
Separate networks for female (**A**) and male (**B**) participants. Non-regularized partial correlation networks containing OTCA, symptoms and pain. Blue edges represent positive and red edges represent negative partial correlations. The OTCA node is placed in the centre of a circle consisting of the remaining variables. To facilitate interpretation, all other edges than those connected to OTCA have been omitted from the graphical presentation. The thickness and brightness of an edge indicate association strength. Legends: OTCA: Paracetamol, ibuprofen or similar, AD1: Felt that everything is a struggle (edge weight females/males: 0.03/0.03), AD2: Had sleep problems (0.06/0.04), AD3: Felt unhappy, sad or depressed (0.01/0.0), AD4: Felt hopeless about the future (0.01/0.0), AD5: Felt stiff or tense (0.02/0.03), AD6: Worried too much about things (− 0.01/− 0.01), AD7: Felt lonely (− 0.01/− 0.02), AD8: Suddenly felt scared for no reason (0.02/0.02), AD9: Felt constant fear or anxiety (0.0/0.01), AD10: Been nervous or felt uneasy (− 0.02/− 0.01), P1: Headache (0.3/0.27), P2: Neck and shoulder pain (0.01/0.02), P3: Joint and muscle pain (0.07/0.1)*,* P4: Abdominal pain (0.1/0.05)

Figure 3 shows the networks for female and male participants. The NCT revealed significant variation in the general network structure (*M* = 0.109, *p* < .001). Overall, the female network was more strongly connected (global strength =7.122) than the male network (6.748; *p* < .001).

Visual inspection revealed that the most prominent edges between OTCA usage and symptoms in the female and male networks were the same as those identified in our previous networks. Statistical comparison of edge weights between OTCA and symptoms identified three edges that were significantly different between females and males (*p <*. 001): “sleep problems” and “hopeless” displayed a stronger connection with OTCA in the female network, whereas “stiff or tense” was more closely linked to OTCA in males.

**Fig. 4:**
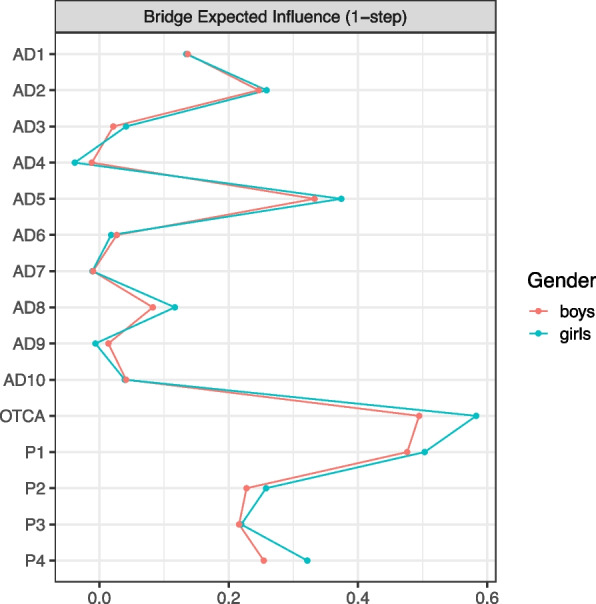
Bridge expected influence (1-step) for females and males. We defined three communities in the female and male network (i.e. OTCA + symptoms + pain) to check for bridge nodes between communities. Bridge expected influence 1-step refers to the sum of the value (positive or negative) of all edges connecting node A to all nodes not in the same community as node A [46]. Legends: OTCA: Paracetamol, ibuprofen or similar, AD1: Felt that everything is a struggle, AD2: Had sleep problems, AD3: Felt unhappy, sad or depressed, AD4: Felt hopeless about the future, AD5: Felt stiff or tense, AD6: Worried too much about things, AD7: Felt lonely, AD8: Suddenlty felt scared for no reason, AD9: Felt constant fear or anxiety, AD10: Been nervous or felt uneasy. Blue edges represent positive and red edges represent negative partial correlations. The OTCA node is placed in the centre of a circle consisting of the remaining variables. The thickness and brightness of an edge indicate association strength, P1: Headache, P2: Neck and shoulder pain, P3: Joint and muscle pain*,* P4: Abdominal pain

The bridge expected influence (BEI) measure for the female and male network is presented in Fig. 4. The most central nodes bridging the three communities in both networks were the same as presented earlier. However, OTCA showed a significantly higher degree of BEI in the female (0.58) than the male (0.49) network (*p <* .05).

### Accuracy and stability checks

The test of network accuracy and stability is presented in the supplementary materials (Fig. S1-S8). Bootstrap analyses showed that the network estimates were highly accurate and stable. The correlation stability (CS)-coefficient for the bridge centrality showed a satisfying level of 0.75 for all networks.

## Discussion

The current study set out to explore the associations between the use of OTCA and distinct symptoms of anxiety and depression in adolescents using network analysis. Results showed associations between OTCA usage and most symptoms. These links remained, albeit with reduced strength, after including pain variables in the network. The strongest associations were observed with “sleep problems”, “stiff or tense”, “everything is a struggle” and “suddenly scared”.

A secondary aim was to examine if the OTCA-symptoms associations differed between genders. We hypothesized females would demonstrate stronger connection between OTCA usage and anxiety and depression symptoms. Comparing the female and male networks demonstrated a significant difference in three edge weights (i.e. association strength between two nodes). The associations between “sleep problems” and OTCA, as well as “hopeless” and OTCA, appeared stronger among females. Conversely, “stiff or tense” was more strongly related to OTCA among males. Furthermore, we found that OTCA exhibited higher bridge expected influence (BEI) in the female network compared to the male network, indicating stronger connections to other communities (symptoms and pain) among females.

### Psychological features of anxiety and depression showed weaker links to OTCA

Our key finding was that overall symptoms characterized by somatic manifestations of anxiety and depression, such as sleep problems and stiffness and tension, were most closely associated with OTCA usage. Anxiety and depression symptoms with clearer cognitive and affective aspects, such as “worrying too much” and “hopeless”, showed no associations with OTCA usage when we adjusted for pain. The absence of a link between these symptoms and OTCA suggests that their relationship was influenced solely by other symptoms in the network, indicating conditional independence. Thus, when controlling for pain symptoms, it appears that some of the core psychological features of anxiety and depression are less relevant for the usage of OTCA.

“Everything is a struggle” and “suddenly scared” were associated with OTCA usage after adjusting for pain. There is no standardized classification of anxiety and depression symptoms into categories such as somatic, cognitive and affective, and several symptoms encompass both psychological and physiological aspects. One could assume that “feeling that everything is a struggle” overlaps with somatic symptoms, such as “feeling low on energy” and “fatigue”, as well as with more cognitive and affective symptoms, such as “loss of interest/joy” [37, 49]. Similarly, “suddenly feeling scared for no reason” is not a distinctly somatic, cognitive or affective symptom. It can involve both physiological reactions and bodily activation, such as heart pounding, trembling and trouble catching one’s breath, and psychological appraisals of being in danger [49].

### On-label symptoms were most prominently related to OTCA usage

On-label symptoms for OTCA usage include headaches, neck and shoulder pain, muscle and joint pain, and abdominal pain. These pain symptoms were expected to be strongly related to the use of OTCA, as it would suggest that the majority of participants use OTCA primarily for its intended purpose. Indeed, including pain nodes in the network revealed substantially stronger associations between OTCA usage and on-label symptoms than between OTCA and off-label symptoms (i.e., anxiety and depression). This is in line with previous research on OTCA usage among adolescents [50].

It is important to note that the associations observed between specific symptoms and OTCA usage in our cross-sectional network do not provide information about the direction of their relationship. We can only speculate on which symptoms may lead to certain behaviors, and vice versa. Moreover, causal relationships can be reciprocal. For example, headaches may lead to OTCA usage, but frequent OTCA usage could also potentially lead to headaches [51, 52] and abdominal issues [53]. We therefore cannot rule out that part of the observed association may be due to OTCA usage having triggered pain. Feedback loops between symptoms may also arise (e.g. worrying ➔ sleep problems ➔ feeling stiff and tense ➔ headaches ➔ OTCA usage ➔ worrying about frequent use) [28]. Capturing temporal or motivational aspects on an individual level would provide more information concerning the directionality of the associations.

Furthermore, the literature shows that the negative side effects of OTCA usage in adolescents are less understood. A recent meta review identified several knowledge gaps and inconsistencies in findings regarding OTCA usage among children up to the age of 18 [54]. Adolescents in particular face an increased risk of unintentional misuse of OTCA [50, 55]. While it is reassuring that the associations between OTCA usage and pain were markedly larger than the associations with anxiety and depression, there is a need for a deeper understanding of the factors that influence decision-making regarding OTCA usage. There could be other variables affecting OTCA usage among adolescents that this study did not control for. For example, it has been suggested that variations in autonomy, inclination for critical risk assessment, and independence from parental influence might contribute to differences in OTCA usage [50, 55]. Additionally, socio-demographic variables and differences in health literacy are among the aspects that have been suggested as possible factors related to OTCA usage [56]. Even though dosage instructions and usage guidelines are readily available on the medicine packages, a sufficient level of basic health literacy is necessary to understand and make use of this information [56, 57]. Future studies should consider including a wider range of variables in their analysis to obtain an even more comprehensive overview of the specific factors related to off-label OTCA usage among adolescents.

### Social pain as a possible mediating factor

Several neurofunctional, cognitive and psychosocial factors are linked to OTCA usage [58]. OTCA may influence how people experience distress, process cognitive discrepancies, and evaluate stimuli in their environment [36, 59]. For example, a single dose of paracetamol in young students can reduce affective reactivity to other people’s positive and negative experiences [34, 35]. Daily usage of paracetamol also seems to reduce behavioural and neurofunctional responses associated with the pain of social rejection [60], and victimization from bullying seems to be related to medicine usage in adolescents [61]. Consequently, it has been suggested that analgesics might be used to alleviate social pain, rather than emotional pain in general [34, 35].

If the observed associations between OTCA usage and AD symptoms were mediated by social pain, we might anticipate a strong relationship between OTCA usage and the symptom “lonely”, which is closely related to social exclusion and has previously been associated with both acute and chronic pain [62]. However, our findings revealed a slight negative relation between the two in all our network analyses. One possible explanation is that individuals who frequently used OTCA experienced reduced feelings of loneliness due to the medication blunting or diminishing such emotions [59, 60]. Social pain, like the unpleasant emotional state of loneliness, shares similarities with the discomfort felt in response to physical pain [63], and research has demonstrated that brain activation patterns in response to social pain overlap significantly with those observed in studies of physical pain [64]. Thus, it seems plausible that adolescents might turn to painkillers when feeling lonely, and that these pills may have an analgesic effect on the social pain. Nevertheless, given the weak relation between OTCA and loneliness observed in our study, regulation of social pain is unlikely to play a central role in explaining off-label OTCA usage in adolescents.

### Males showed stronger relations between somatic symptoms and OTCA usage

Several studies point to a stronger relation between OTCA usage and anxiety and depression among females compared to males [2, 23]. Thus, we hypothesized that gender-specific networks would demonstrate differences in edge weights, with females exhibiting stronger connections between OTCA usage and symptoms of anxiety and depression.

In line with our hypothesis, results revealed significant distinctions in edge weights between females and males, though only for three of the edges. Among these, two exhibited greater strength within the female network: the connections between “sleep problems” and OTCA, and “hopeless” and OTCA, were notably stronger compared to those in males. Conversely, the association between “stiff or tense” and OTCA was more pronounced among males. Moreover, results showed that OTCA displayed a higher BEI within the female network, suggesting stronger ties between OTCA and other communities (symptoms and pain) among females compared to males. While we did observe a trend in the direction we anticipated, we would expect to see more distinct and pronounced associations between OTCA and symptoms within the female network, considering the remarkable gender differences in numerous other aspects related to anxiety, depression and OTCA usage.

Research on gender differences in physical pain perception tends to show that, compared to males, females have lower pain thresholds, higher pain ratings and reduced tolerance for painful stimuli [24]. Considering this, one might expect clearer somatic symptoms such as stiffness and tension to trigger more OTCA usage in females, as women might more easily find these symptoms painful. Nonetheless, our findings yielded the opposite result; “stiff or tense” was more closely related to OTCA usage in males than females. This could be explained by the fact that males seem to obtain a greater analgesic effect from OTCA than females [25], thus making them turn more easily to analgesics for somatic complaints.

That OTCA are less effective in reducing physical pain in females than in males, while females are highly overrepresented among high-frequency OTCA users, highlights the need for more knowledge on off-label usage across genders. A study by Vangelisti et al. [65] sought to assess gender differences in social pain experiences in response to ibuprofen. Results revealed that females in the ibuprofen group reported reduced social pain on several tasks, while males in the same group exhibited the opposite pattern: the ibuprofen group experienced *more* social pain than the control group. If the ibuprofen’s effect on social pain was mainly physiological, we would expect to see a greater effect in the male group, and not the other way around. The researchers therefore suggested a social cognitive explanation: ibuprofen reduced females’ sensitivity to social pain due to its pain-relieving effects, while it disrupted males’ natural inclination to suppress emotional pain. If, indeed, OTCA make females feel better, and males feel worse, at least when it comes to the social aspects of pain, this could help explain why OTCA usage is much more common among females. However, our results showed that sleep problems and feelings of hopelessness were more closely related to OTCA usage in females than males, while the rest of the symptoms, including feelings of loneliness, showed no differences.

Though it is compelling to dwell on the highlighted disparities between the gender-specific networks in our study, it is worth noting that the observed differences are relatively small. A visual inspection of the two networks revealed that the associations’ structure and strength did not differ greatly. Given the highlighted research on gender differences, one might wonder why the observed connections are not even more pronounced in females compared to males. It is plausible that this can be ascribed to females experiencing more pain than males, resulting in increased OTCA usage, while the proportion of off-label usage remains similar between the two groups. More studies like Vangelisti’s, with strict designs and randomization into groups, should be carried out to gain more information on how OTCA directly influence different emotional, social, cognitive and somatic factors across genders.

### Strengths, limitations and implications for future research

To our knowledge, the current study is to date the only network analysis assessing OTCA usage and symptoms of anxiety and depression in adolescents. The sample was population-wide and thus representative of adolescents in Norway. Another strength of this study was its focus on the individual symptoms of anxiety and depression, rather than diagnostic categories. This approach allowed for a more detailed examination of specific symptoms that might be particularly central in explaining the association between symptoms and OTCA usage in adolescents, and across genders.

Including more symptoms in the network, such as all 58 items from the original HSCL [37], could provide us with a more comprehensive and nuanced understanding of which symptoms, and groups of symptoms, are most central in explaining off-label OTCA usage among adolescents. Notably, the depression inventory included in our survey data did not include any measure of anhedonia (lack of positive affect), a core symptom of depression known to represent a distinct feature [66] and correlate with symptom severity across various mental health conditions [67]. Thus, our study cannot draw conclusions regarding this central aspect of depression. Further studies should make use of instruments that capture depression and anxiety more broadly, including specific assessments for anhedonia, such as the Inventory of Depressive Symptomatology [68] or the Patient Health Questionnaire [69].

In the current study, conducted on Norwegian adolescents, it is reasonable to assume that OTCA usage primarily comprises paracetamol (acetaminophen) and ibuprofen. However, exploring other pain relievers containing different active ingredients, not restricted solely to OTCA, would provide more valuable insights as to how analgesics relate to psychological and physiological pain across genders. For instance, existing research suggests that the gender differences in the effects of morphine are more pronounced than those observed with OTCA [70].

As our research suggests a gender difference in on- and off-label OTCA usage in certain specific domains among adolescents, it should be of high priority to further investigate and gain a deeper understanding of how these differences manifest in their daily lives. This knowledge could potentially contribute to reducing unnecessary and frequent use of OTCA. Moreover, it would be intriguing to compare various subgroups within the population, such as high-frequency versus low-frequency OTCA users and those with many symptoms versus those with few. Further, measuring OTCA usage over time and in controlled settings, would provide a more comprehensive understanding of the complex relationship and the causal directions between OTCA usage and symptoms of anxiety and depression.

## Conclusion

Our research provides a thorough examination of the complex associations between OTCA usage and specific symptoms of anxiety and depression in adolescents. Our primary aim was to investigate these potential links using a network approach. We found that OTCA usage was most strongly associated with on-label symptoms, such as headache, neck and shoulder pain, muscle and joint pain and abdominal pain. However, OTCA usage was also associated with symptoms of anxiety and depression. Most prominent were the associations with sleep problems, stiffness and tension, feeling that everything is a struggle, and feeling suddenly scared for no reason. Symptoms marked by somatic manifestations of psychological distress exhibited more robust associations with OTCA usage compared to other symptoms of anxiety and depression.

Our secondary aim was to examine whether the associations between OTCA usage and specific symptoms varied by gender. As hypothesized, females demonstrated stronger associations between OTCA usage and anxiety and depression symptoms than males. Conversely, clear somatic symptoms seemed to be more highly associated with OTCA usage in males compared to females. However, the overall pattern remained consistent across both genders.

### Supplementary Information

**Supplementary Material 1.**

## Data Availability

The datasets analyzed during the current study are not publicly available due to lack of consent to sharing individual data. Meta data is available from the corresponding Young Data Survey on reasonable request. Data and material are stored at Oslo Metropolitan University P.O. Box 4, St. Olavs plass. N-0130 OSLO, Norway.

## Abbreviations

OTCA: Over-the-counter analgesics
HSCL: Hopkins symptom checklist
GGM: Gaussian graphical models
BEI: Bridge expected influence
NCT: Network comparison test
