# Supplementary material for: Associations between over-the-counter analgesics usage and symptoms of anxiety and depression in adolescents: a network analysis
Source: BMC Psychiatry. 2024 May 15;24:366. doi: 10.1186/s12888-024-05802-3 (PMC11095036; doi:10.1186/s12888-024-05802-3)
Supplement: Supplementary file 1 — Supplementary Material 1. [file 12888_2024_5802_MOESM1_ESM.docx]

**Supplementary Figures**


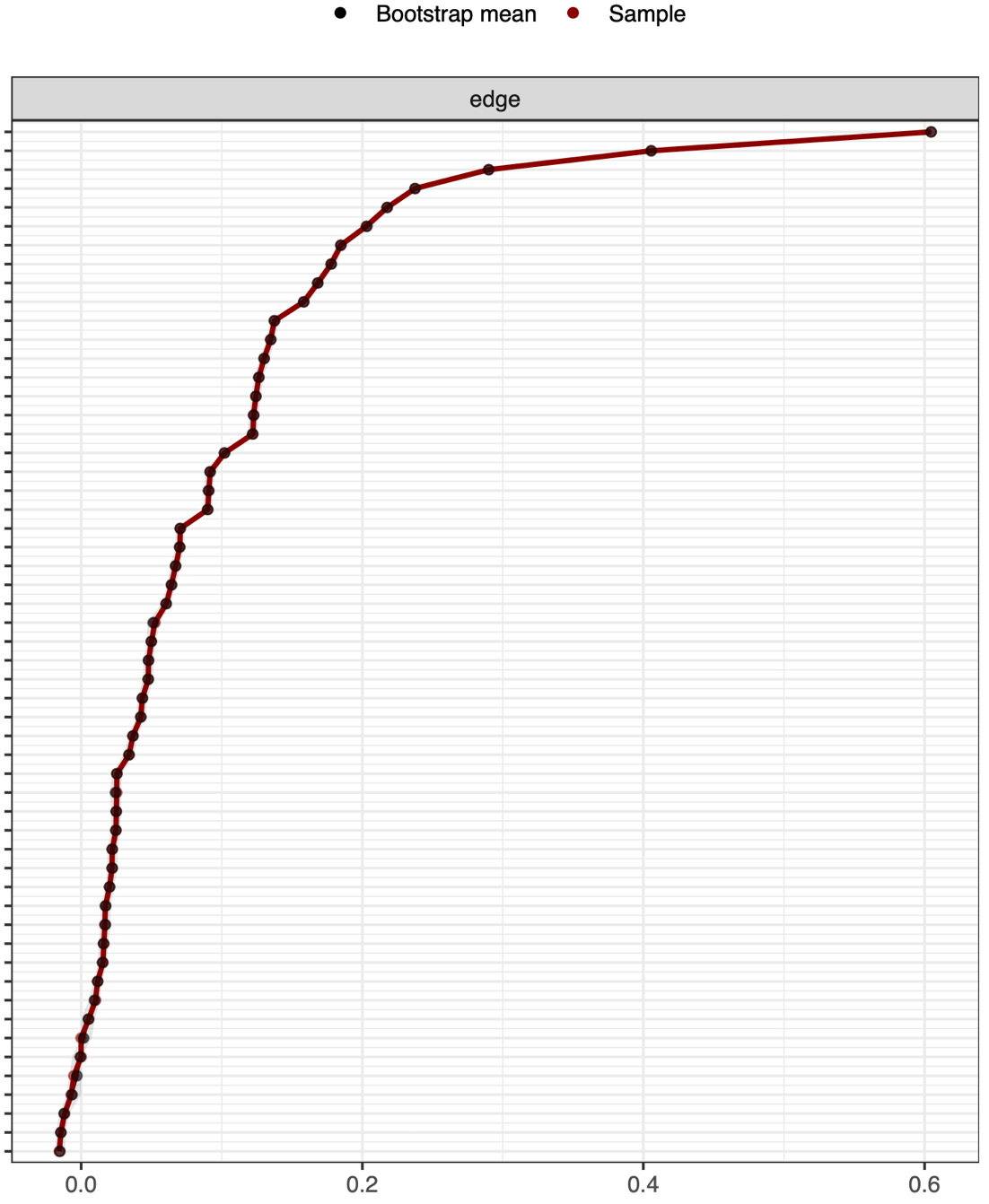


*Supplementary Figure 1:* Accuracy and stability of the distribution of bootstrap means in Network 1.


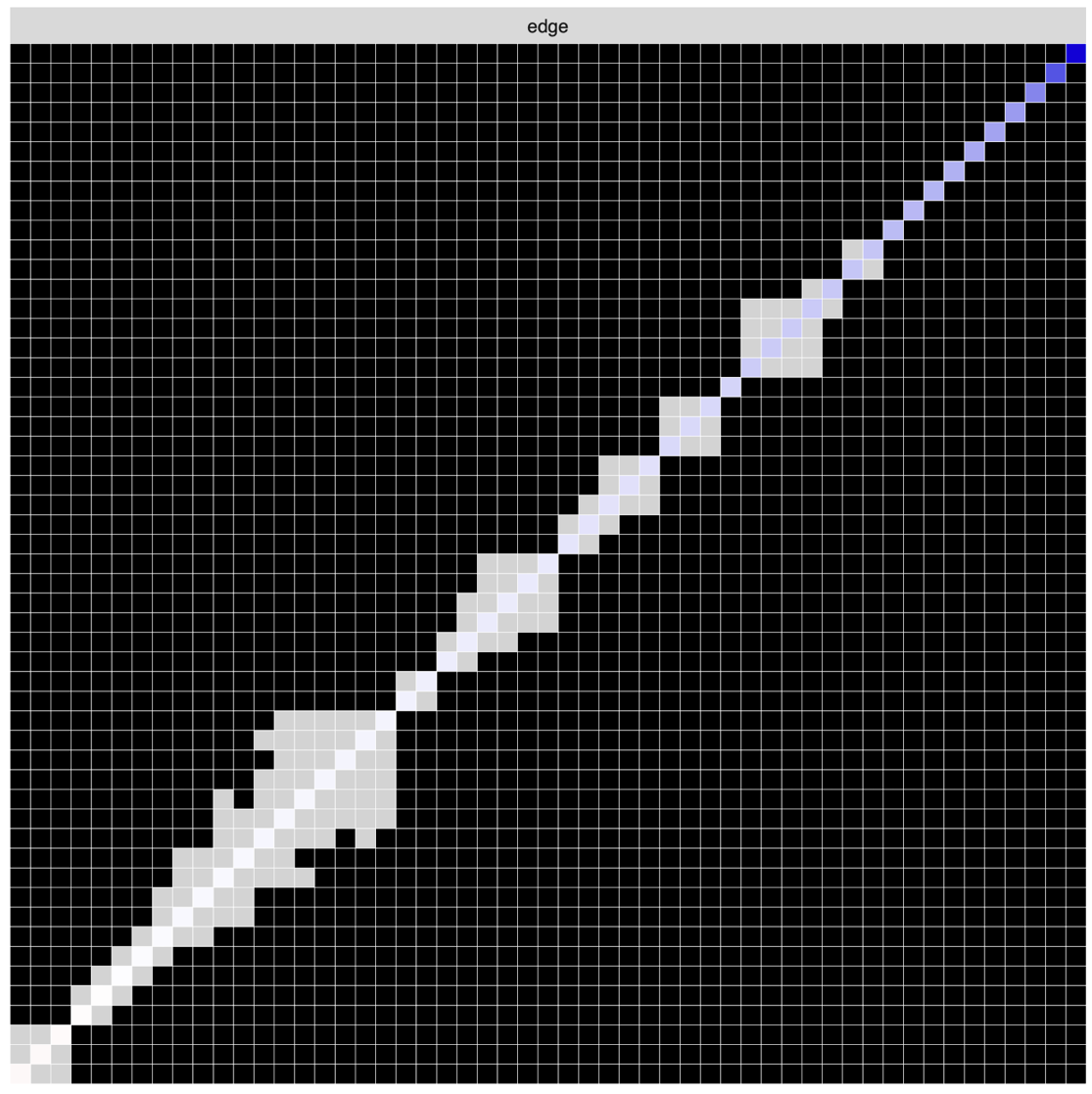


*Supplementary Figure 2:* Accuracy and stability of edge-estimates in Network 1.

*
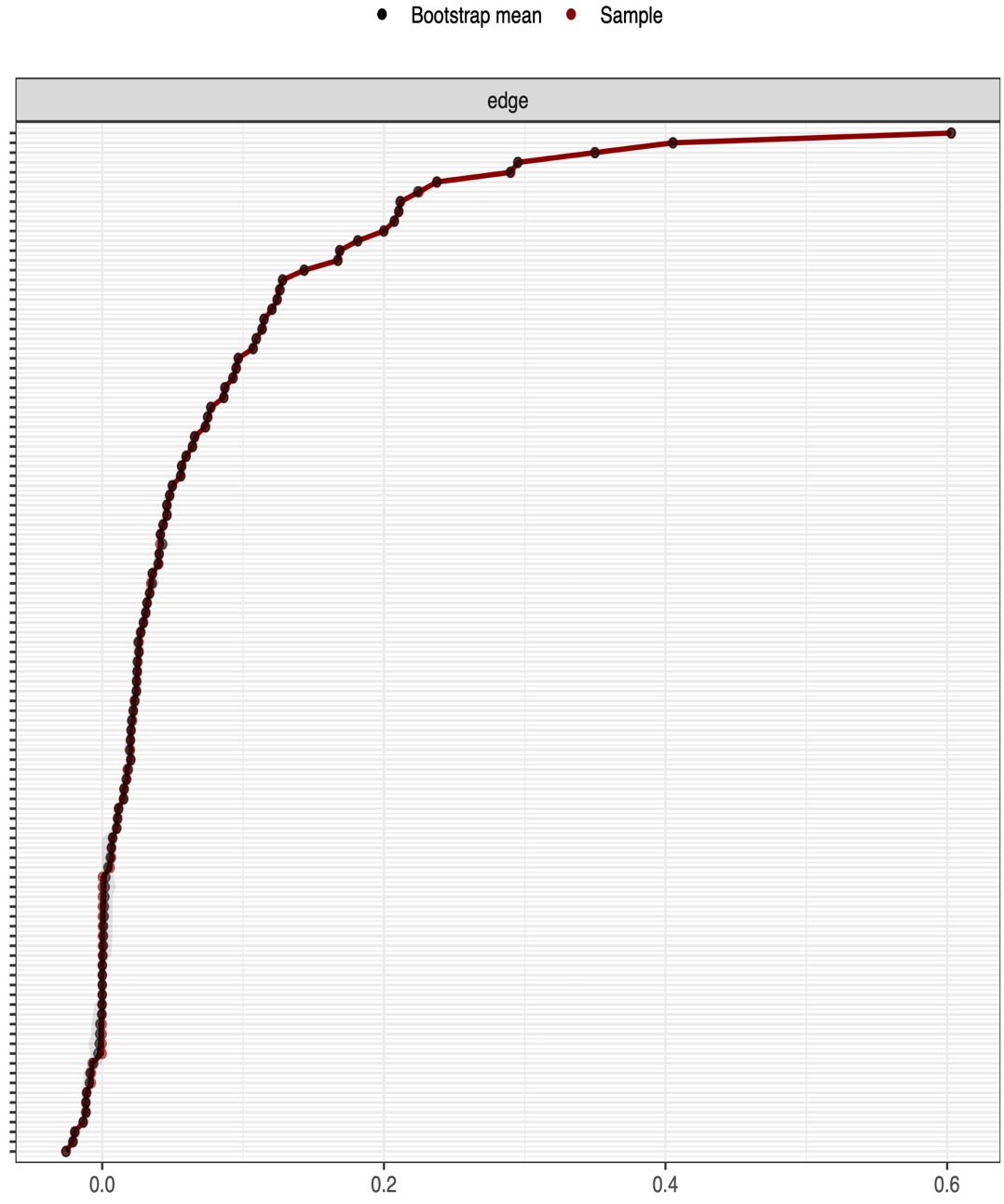
*

*Supplementary Figure 3:* Accuracy and stability of the distribution of bootstrap means in Network 2.


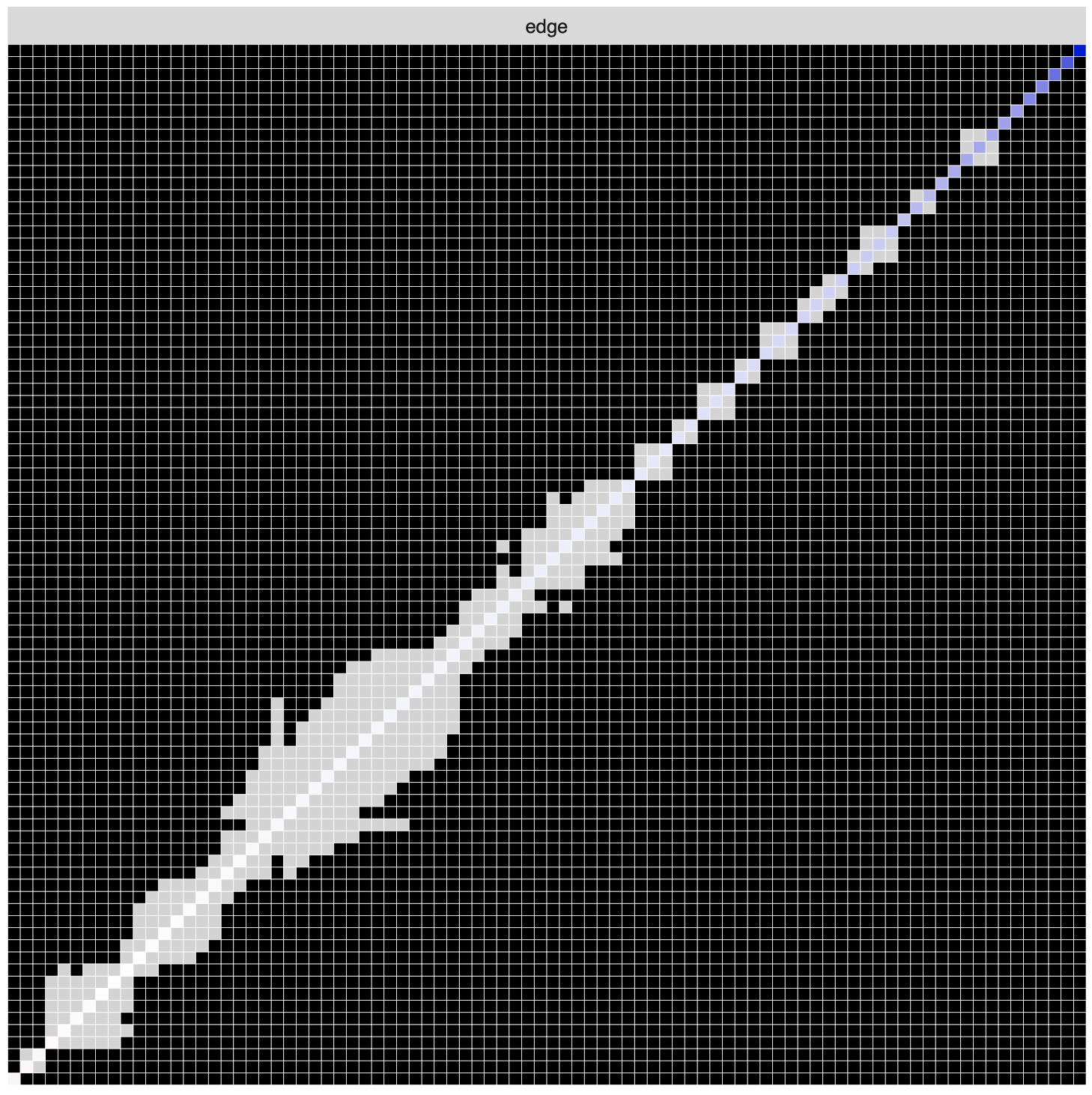


*Supplementary Figure 4:* Accuracy and stability of edge-estimates in Network 2.

*
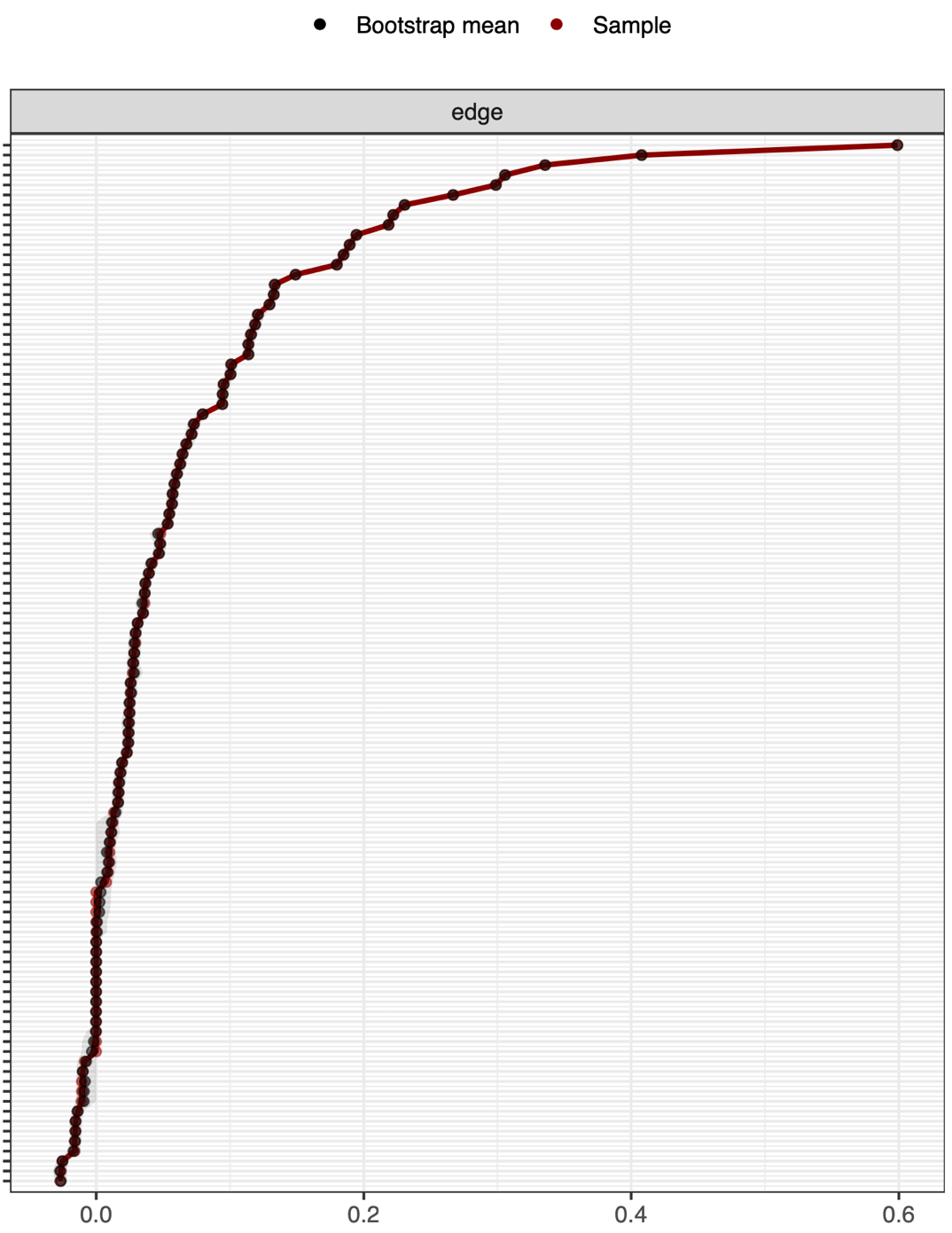
*

*Supplementary Figure 5:* Accuracy and stability of the distribution of bootstrap means in the female network.


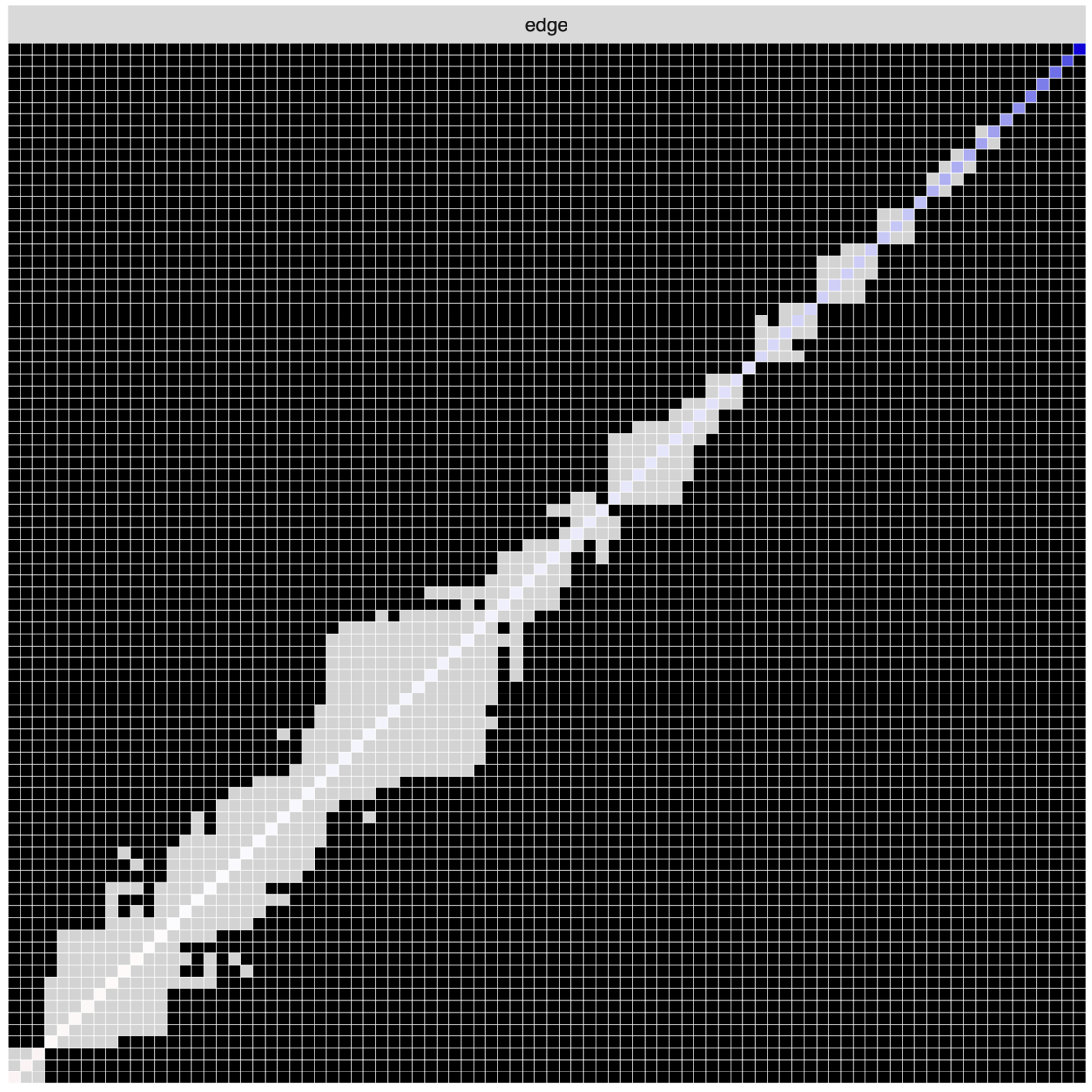


*Supplementary Figure 6:* Accuracy and stability of edge-estimates in the female network.

**
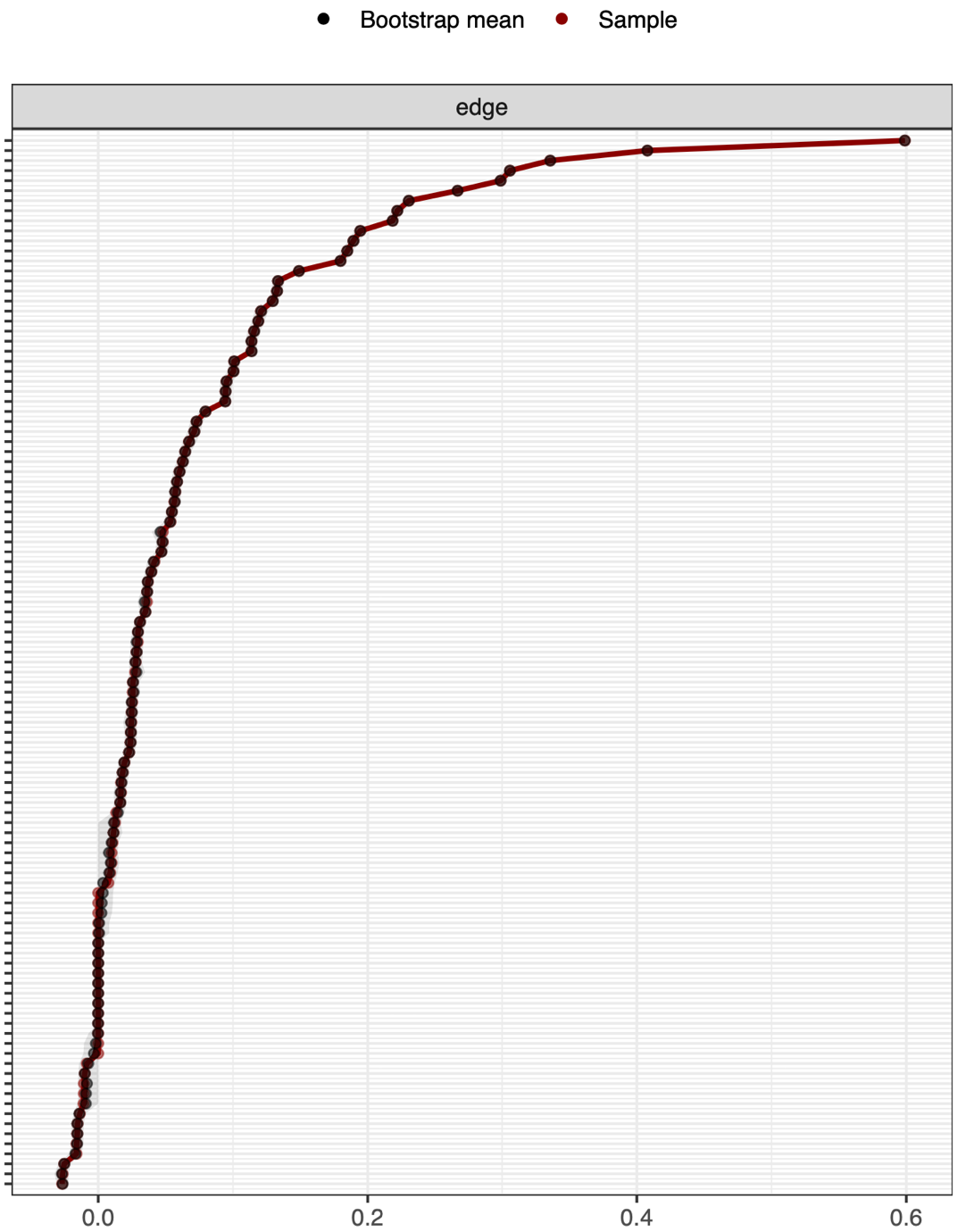
**

*Supplementary Figure 7:* Accuracy and stability of the distribution of bootstrap means in the male network.

**
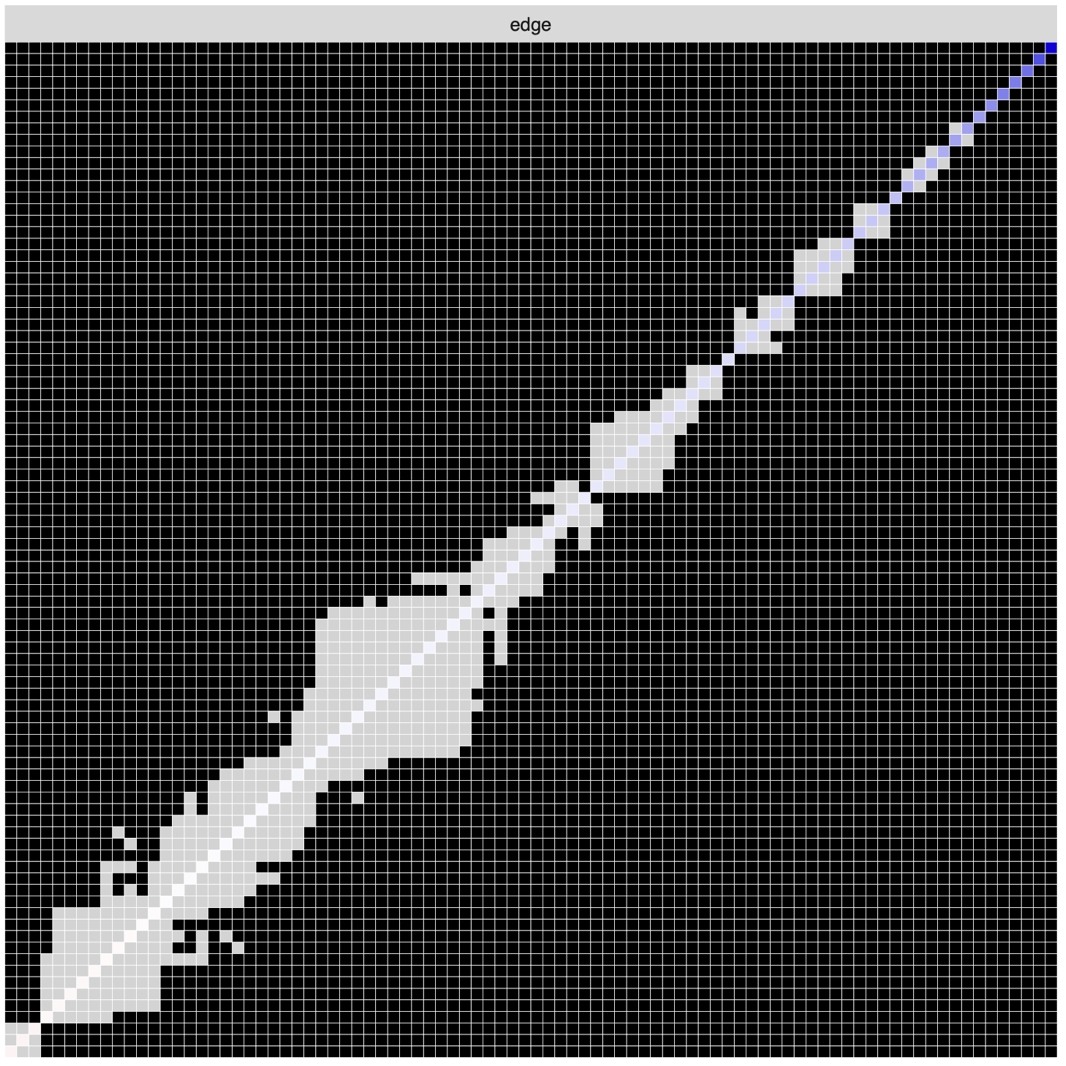
**

*Supplementary Figure 8:* Accuracy and stability of edge-estimates in the male network.


*Supplementary Figure 9:* Expected influence in Network 1, presented in raw scores.

*Supplementary Figure 10:* Expected Influence in Network 2, presented in raw scores.

*Supplementary Figure 11:* Expected influence in the female and male networks, presented in raw scores.

*Supplementary Figure 12:* Network 1 with all edges visualized.

*Supplementary Figure 13:* Network 2 with all edges visualized.

*Supplementary Figure 14:* Female network with all edges visualized.

*Supplementary Figure 15:* Male network with all edges visualized.
